# Growth Differentiation Factor 15 (GDF-15) Levels Associate with Lower Survival in Chronic Kidney Disease Patients with COVID-19

**DOI:** 10.3390/biomedicines10123251

**Published:** 2022-12-14

**Authors:** Andrea Galassi, Paola Ciceri, Valeria Bono, Lorenza Magagnoli, Matteo Sala, Luisa Artioli, Roberta Rovito, Mohamad Hadla, Vaibhav Yellenki, Antonella D’Arminio Monforte, Camilla Tincati, Mario Cozzolino, Giulia Marchetti

**Affiliations:** 1 Department of Health Sciences, Renal Division, University of Milan, ASST Santi Paolo e Carlo, 20142 Milan, Italy; 2 Department of Health Sciences, Clinic of Infectious Diseases, University of Milan, ASST Santi Paolo e Carlo, 20142 Milan, Italy

**Keywords:** CKD, COVID-19, GDF-15, mortality

## Abstract

A cytokine storm drives the pathogenesis of severe COVID-19 infection and several biomarkers have been linked to mortality. Chronic kidney disease (CKD) emerged as a risk factor for severe COVID-19. We investigated the association between selected biomarkers and mortality in 77 patients hospitalized for COVID-19, and whether they differ in patients with eGFR higher and lower than 45 mL/min. The association between patients’ characteristics, plasma biomarkers and mortality was conducted by univariate logistic regression models and independent predictors of mortality were then used to create a multivariate prediction model through Cox regression. Patients with lower eGFR had a significant increase of GDF-15, CD-25 and RAGE, with higher plasma levels in non-survivors and in patients who needed ventilation. At univariate analysis, low and mid-low GDF-15 quartiles (<4.45 ng/mL) were associated with lower mortality risk, while mid-high and high quartiles (>4.45 ng/mL) were associated with higher mortality risk. Independent association between GDF-15 quartiles and mortality risk was confirmed in the Cox model and adjusted for eGFR, age, fever and dyspnea (HR 2.28, CI 1.53–3.39, *p* < 0.0001). The strength of the association between GDF-15 quartiles and mortality risk increased in patients with lower compared to higher eGFR (HR 2.53, CI 1.34–4.79 versus HR 1.99, CI 1.17–3.39). Our findings may suggest a further investigation of the effect of GDF-15 signaling pathway inhibition in CKD.

## 1. Introduction

By October 2020, severe acute respiratory syndrome coronavirus 2 (SARS-CoV-2), causing the coronavirus disease 2019 (COVID-19) pandemic, had infected millions of people, causing millions of deaths [1]. A key unmet clinical need is the earlier and more precise identification of subjects at a higher risk of severe disease, exploring the need to investigate the disease-associated factors to individuate patients with COVID-19 poor prognosis. Besides the disease per se, several comorbidities are associated with the severity of COVID-19 infection, also implying a need to investigate the potential impact of medications commonly used [2,3].

Recently, chronic kidney disease (CKD) emerged as one of the strongest risk factors for severe COVID-19 [4,5,6,7]. Indeed, patients with advanced CKD are at an increased risk of mortality from several causes, led by cardiovascular disease (CVD) and infections [8,9]. It is very important to underline the relevancy of CKD to the course of COVID-19 disease since the impairment of renal function is often missed by physicians. 

COVID-19 is characterized by a cytokine storm that contributes to the development of endothelial vascular dysfunction, which can lead to acute respiratory distress syndrome, multi-organ failure and finally, death [10]. Several biomarkers are deregulated in the disease course. An emerging factor is the growth and differentiation factor 15 (GDF-15), which belongs to the transforming growth factor-beta (TGF-beta) superfamily of proteins. It has been demonstrated that GDF-15 has a pivotal role in the development and progression of diseases such as CKD [11], congestive heart failure (CHF) [12] and chronic pulmonary obstructive disease (COPD) [13] because of its role as a metabolic regulator [14]. In COVID-19, GDF-15 activity represents a strong predictor of poor outcomes in critically ill patients acting as a central mediator of inflammation [15]. Due to the role of CKD as a major risk factor for severe COVID-19, the research interest on the inflammatory response to SARS-CoV-2 in CKD patients is continuously growing in order to provide clues on the pathogenesis of COVID-19 and on successful treatments in CKD patients [16]. Furthermore, the association between GDF-15 and COVID-19 in CKD patients has been poorly investigated.

The present study explores the association between GDF-15 and in-hospital mortality among CKD patients hospitalized for COVID-19.

## 2. Methods and Materials

### 2.1. Study Design and Population

This was a retrospective observational study conducted on patients hospitalized due to COVID-19 infection and admitted in the ward of Tropical and Infectious Diseases at San Paolo Hospital in Milan (Italy) from February to September 2020. 

### 2.2. Data Collection

Data on demographics, medical history and clinical status were taken from electronic clinical charts and recorded on the online database application REDCap; the data, therefore, was collected from it for the purpose of the present research. Estimated glomerular filtration rate (eGFR) was assessed by a CKD-EPI formula at hospital admission and patients were stratified in two groups (eGFR ≥ 45 mL/min/1.73 m^2^ or eGFR < 45 mL/min/1.73 m^2^). Data on mortality and on the length of stay were collected as part of the study protocol. 

### 2.3. Plasma Cytokine Quantification

Peripheral blood samples collected at admission were centrifuged for 15 min at 2.500 rpm. Plasma was then harvested and stored at −80 °C. Plasmatic levels of the following 20 biomarkers were thereafter assessed by Luminex technology and ELISA assay, according to the manufacturer’s instructions: GDF-15, CD-25, receptor for advanced glycation end products (RAGE), interleukine-6 (IL-6), interleukin-7 (IL-7), interleukin-18 (IL-18), interleukine-6 receptor (IL-6R), tumor necrosis factor alpha TNFa, tumor necrosis factor receptor 1 (TNFR I), tumor necrosis factor receptor 2 (TNFR II), leukemia inhibitory factor (LIF), Fas, YKL-40, pentraxin-3 (PTX-3), platelet derived growth factor—AA (PDGF-AA)—vascular endothelial growth factor (VEGF), a-1-acid glycoprotein (a1-AGP), TNF-related apoptosis-inducing ligand (TRAIL), kynurenine and tryptophane. 

### 2.4. Statistical Analysis

Categorical variables were reported as rates (%). Continuous variables were reported as mean ± standard deviation and median [interquartile range], according to the normality of distribution assessed by the Shapiro-Wilk test. Comparisons between categorical and continuous variables were performed by Fisher’s exact test and Mann–Whitney U test, respectively. Demographic, clinical and biochemical characteristics at baseline were stratified according to eGFR (higher-equal vs lower than 45 mL/min/1.73 m^2^). Plasma biomarkers were also stratified according to survival and the need of non-invasive ventilation (NIV). Linear correlation between biomarkers and eGFR was assessed by a Pearson correlation test.

The association between patients’ characteristics, plasma biomarkers and mortality was first conducted by univariate logistic regression models. Independent predictors of in-hospital mortality (*p* < 0.05) were then investigated by Cox proportional hazard models selected by stepwise procedure. Predictors included at the first step were arbitrarily selected based on the significance of their univariate association with mortality (for clinical characteristics) and with eGFR, NIV and mortality (for biochemical markers). Independency from basal renal function was tested by a forced inclusion of eGFR into the first step of model selection. The proportional hazards assumption was checked by Schoenfeld residuals test for both the single covariates and the whole model. The association between single GDF-15 quartiles and mortality was investigated by univariate logistic regression models. Survival curves for GDF-15 quartiles were plotted by the Kaplan-Meier method. Nonlinear association between GDF-15 and survival was modelled by polynomial splines in all the patients and in eGFR subgroups. A sensitivity analysis was performed by excluding outliers for GDF-15. Furthermore, as reduced eGFR at admission might be due to either CKD (known or unrecognized) or acute kidney injury, the association between GDF-15 and mortality risk was also assessed in the subset of patients with eGFR < 45 mL/min/1.73 m^2^ and known CKD reported in their medical history as a sensitivity analysis. The *p*-value for significance was set at <0.05.

Analysis was conducted by R package version 4.1.1.

## 3. Results

Seventy-seven patients aged 79 {70–86} years were enrolled. Patients’ characteristics are presented in Table 1A,B. In the overall population median, eGFR was 48.4 mL/min/1.73 m^2^. Twenty (26%) patients reported a history of CKD with 4 (5%) patients receiving maintenance hemodialysis. Thirty-three (43%) patients presented eGFR < 45 mL/min/1.73 m^2^. A history of cardiovascular disease (CVD) was highly prevalent (49%), including patients affected by CHF (14%), myocardial infarction (22%) and arrhythmias (19%). Diabetes and COPD were reported in 25 (32%) and 9 (12%) patients, respectively. The most frequent symptoms at admission were fever (69%), cough (34%) and dyspnea (56%). Pneumonia and acute respiratory distress syndrome were documented in 67 (87%) and 36 (47%) patients, respectively. Forty-two (55%) patients required NIV, while only 2 (3%) were admitted to an intensive care unit (ICU). The specific treatments most frequently prescribed included heparin (75%), hydroxychloroquine (74%) and steroids (22%). The median time from symptom onset to hospitalization was 4 days (IQR 2–8). A 45% in-hospital mortality rate was observed. Median time from admission to death or dis-charge was 18 {11–35} days. Patients with eGFR < 45 mL/min/1.73 m^2^ presented higher prevalence of CKD, diabetes and higher neutrophil count. Lower eGFR was not associated with any other significant difference in baseline characteristics, clinical severity and in-hospital mortality rate.

**Figure 1:**
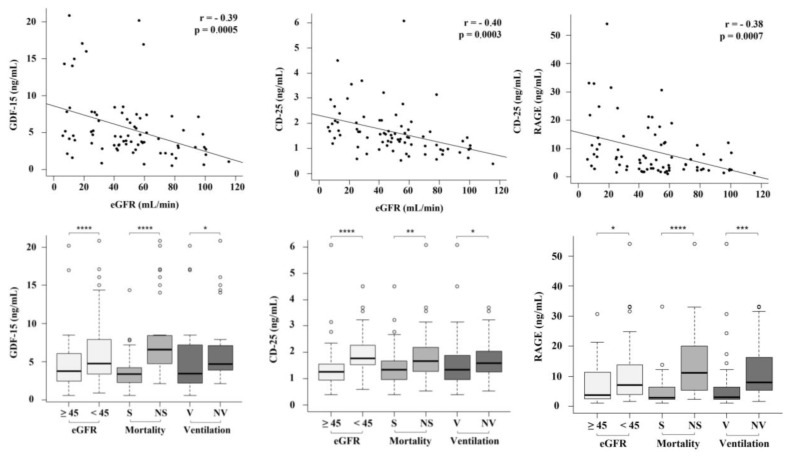
Association between GDF-15, CD-25 and RAGE with eGFR, ventilation and mortality. Abbreviations: eGFR, estimated glomerular filtration rate; GDF-15, growth and differentiation factor 15; RAGE, receptor for advanced glycation end products; S, survivors; NS, non survivors; NV, non-ventilated; V, ventilated; <45, eGFR < 45 mL/min/1.73 m^2^; >45 mL/min, eGFR > 45 mL/min/1.73 m^2^; *, *p* < 0.05; **, *p* < 0.01; ***, *p* < 0.001; ****, *p* < 0.0001.

GDF-15, CD-25 and RAGE resulted in the unique plasma biomarkers significantly associated with eGFR, a need of NIV and mortality out of the 20 tested molecules (Table S1). Plasma levels of these biomarkers were negatively associated with basal eGFR (Figure 1). Plasma concentrations of GDF-15, CD-25 and RAGE were significantly higher in deceased patients and in those receiving NIV (Figure 1).

**Figure 2:**
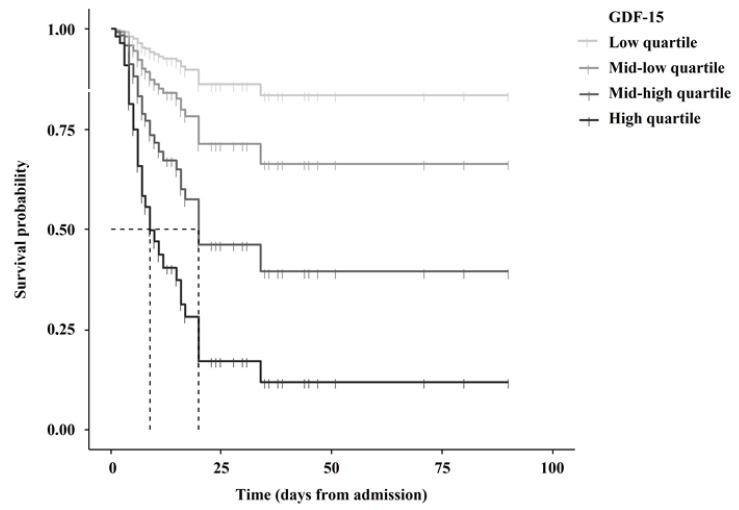
Association between GDF-15 quartiles and survival.

Patients with an age > 75 years (*p* = 0.005), fever (*p* = 0.005), dyspnea (*p* = 0.003) and P/F < 300 (*p* = 0.034) were significantly associated with mortality at univariate analysis. Survival curves stratified according to GDF-15 quartiles are presented in Figure 2. In a multivariate Cox regression model, each increase in GDF-15 quartiles was associated with a 128% increased mortality risk [HR 2.28 (1.53–3.39, 95% CI), *p* < 0.001] independent from basal eGFR and the aforementioned predictors (Table 2). CD-25 and RAGE were excluded from the model by a stepwise selection procedure. Stronger association between GDF-15 and mortality was descriptively observed among patients with eGFR < 45 mL/min/1.73 m^2^ [HR 2.54 (1.34–4.79), 95% CI] compared with a higher eGFR strata [HR 1.99 (1.17–3.39, 95% CI)]. Results were unchanged in the sensitivity analysis after the exclusion of 8 outlying observations for GDF-15 (Table S2) and dialysis patients (Table S3). 

**Figure 3:**
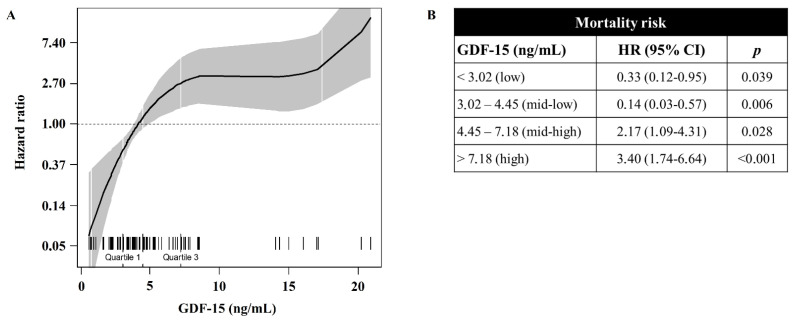
Univariate association between GDF-15 and mortality risk. (**A**) Cubic spline analysis revealing nonlinear association between GDF-15 and mortality risk: the solid line represents the HR according to GDF-15 level, the gray area represents the 95% CI, ticks in the lower part of the figure represent each observation. (**B**) Univariate association between GDF-15 quartiles and mortality risk; each quartile was compared with 3 other quartiles as a whole comparator.

**Figure 4:**
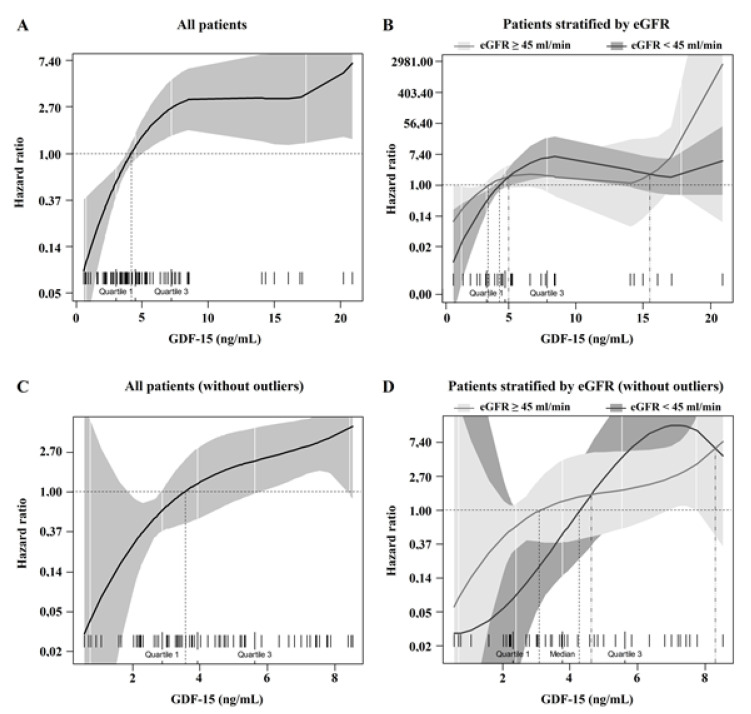
Non-linear association between continuous GDF-15 and mortality risk, adjusted for eGFR, age, fever, dyspnea and P/F. The solid line represents HR according to GDF-15 level. The colored area represents 95% CI, ticks in the lower part of the figure represent each observation. (**A**) All patients of the whole study cohort. (**B**) Patients stratified according to eGFR strata. (**C**) All patients, excluding outliers for GDF-15. (**D**) Patients stratified by eGFR, excluding outliers for GDF-15.

Univariate polynomial splines revealed a nonlinear association between GDF-15 and survival (Figure 3A). At univariate analysis, first and second GDF-15 quartiles were singularly associated with a lower mortality risk [HR 0.33 (0.12–0.95, 95% CI) and HR 0.14 (0.03–0.57, 95% CI), respectively] (Figure 3B). On the other hand, third and fourth GDF-15 quartiles were singularly associated with an increased mortality risk [HR 2.13 (1.09–4.31, 95% CI) and 3.4 (1.74–6.64, 95% CI), respectively]. Protective and harmful associations between GDF-15 and mortality were observed for circulating levels below and beyond the median (4.45 ng/mL), respectively, after adjustment for age, fever, dyspnea, P/F and eGFR (Figure 4A). Both the protective and the harmful associations between GDF-15 and mortality were descriptively more pronounced among patients with eGFR < 45 mL/min/1.73 m^2^ (Figure 4B). The trend was confirmed after the exclusion of GDF-15 outliers (Figure 4C,D). In the subset of patients with eGFR < 45 mL/min/1.73 m^2^ and known CKD reported in their medical history (18 patients, 10 of whom died), we found a linear and positive association between GDF-15 and mortality risk in the univariate Cox regression [HR 1.13 (1.01–1.27, 95% CI) for every 1 ng/mL increase in GDF-15 level], although the small sample size and low number of events did not allow us to investigate this association in multivariate models.

## 4. Discussion

Since the initial description of COVID-19 at the end of 2019 [17,18], over 250 million confirmed cases of COVID-19 have been reported to date, with more than 6 million deaths worldwide (World Health Organization, April 2022). While the majority of patients develop mild to moderate COVID-19, severe disease has been shown to occur in about 10–15% of infected individuals with a critical disease [19]. 

Several clinical and epidemiological factors have been associated with the development of severe COVID-19 and include older age, obesity and dysmetabolic co-morbidity, hypertension and immune depression [1,2,3]; however, a detailed profile of the pathogenetic pathways associated with the worst disease outcome is still largely elusive.

Among clinical factors associated with disease severity, CKD retains a high impact on the poor outcomes of COVID–19, underlining the importance of identifying strategies to prevent SARS-CoV-2 infection in CKD [20]. Undoubtedly, infections, sepsis and bacteremia represent major causes of morbidity and mortality in renal patients [21]. Moreover, infections in CKD patients cause a longer duration of hospitalization and a higher mortality rate from pneumonia [22,23]. Therefore, the choice of renal replacement treatment in advanced CKD patients, with techniques able to efficiently remove uremic toxins and reduce infection risk [24], remains important.

Indeed, a condition known as cytokine release syndrome has been described as a hallmark of aggressive COVID-19 that consists of the uncontrolled release of both pro- and anti-inflammatory cytokines, and that in turn is associated with tissue damage and dysfunctional and delayed immune response [25]. In the early phase of the pandemic, several biomarkers of the importance in dictating COVID-19 severity were first identified in case series of patients hospitalized with COVID-19 [2,3,4]. Furthermore, numerous inflammatory and cardiovascular biomarkers were assessed in association with outcome and were identified as particularly strong prognostic markers [26]. 

Because CKD has been associated with COVID-19 severity, we hereby sought to investigate the clinical role of several biomarkers in COVID-19 outcomes in the setting of CKD patients hospitalized for COVID-19.

Our bio-bank study of unselected, consecutive patients hospitalized with COVID-19 provides important insights to these associations, given that our design alleviates the risk of selection bias. Interestingly, GDF-15 was the only cytokine to be retained in the regression model for predicting mortality risk in patients with eGFR < 45 mL/min/1.73 m^2^.

GDF-15 is a member of the TGF-beta superfamily and patrols immunotolerance during pregnancy, as witnessed by its high placental expression [27]. GDF-15 is secreted as a 25 kDa dimer [28] in several other organs, including the kidney, lungs, heart, brain, lymph nodes, bladder and prostate [27,29], where it is endowed with the potential to mediate immune response, inflammation tissue tolerance, energy homeostasis and body weight regulation [30]. Notably, multiple cell lines participate in GDF-15 synthesis as macrophages, endothelial cells, epithelial cells, vascular smooth muscle cells, adipocytes and cardiomyocytes [15]. Although GDF-15 expression is mainly quiescent outside of reproductive organs, it increases in several conditions of tissue damage triggered by inflammatory and oxidative stimuli [31,32,33]. GDF-15 has been postulated to enhance the ability of tissues to control the inflammatory insult through metabolic adaptation [34] as well as control immune cell infiltration [27]. However, GDF-15 was also associated with the severity and progression of acute-as-chronic diseases involving renal [11], cardiovascular [35,36], respiratory [13] and immune systems [37] in humans. 

Renal expression of GDF-15 was documented in tubular cells, where it is hypothesized to enhance the protective response against renal damage [11,38]. However, observational studies reported a direct association between GDF-15 and an increased risk of incident CKD [39] and CKD progression [11]. GDF-15 resulted in an independent predictor of mortality in stage-3 CKD [40,41], as well as in dialysis patients [42]. Furthermore, GDF-15 emerged as a promising risk factor in cardiorenal syndrome. GDF-15 was associated with the risk of CHF in renal patients and predicted mortality in CHF [40,43]. Interestingly, although GDF-15 is expressed in cardiomyocytes, the majority of circulating GDF-15 in patients with CHF was postulated to be of renal origin, secondary to kidney injury induced by venous congestion. Nonetheless, pulmonary epithelial and endothelial cells express GDF-15 under stimulation by hypoxia [44], cigarette smoking [45] and shear stress [46]. In vitro and animal models recently identified GDF-15 as an amplifier of lung inflammation during viral infections [47], therefore representing a major pathogenetic mechanism of susceptibility and disease severity in patients with already damaged airways. 

COVID-19 represents a peculiar condition of systemic inflammation with multi-organ involvement, including pulmonary, cardiac and renal damage, which is often responsible for life threatening implications [48]. GDF-15 integrates information on cellular oxygenation, inflammatory response and cardio-renal dysfunction, which are all key mechanisms in COVID-19 pathophysiology, suggesting GDF-15 as an ideal candidate as a prognostic marker in COVID-19.

GDF-15 has been associated with poorer respiratory function, disease severity and mortality among hospitalized patients hospitalized due to SARS-CoV-2 infection [30,49,50]. However, the association of GDF-15 with disease severity and mortality is mainly unexplored in renal patients. To date, a unique study by Gisby et al. identified GDF-15 as a relevant biomarker of COVID-19 severity among 55 dialysis patients out of 203 tested molecules [51].

To our knowledge, this is the first study designed to investigate GDF-15 prognostic value in non-dialysis renal patients hospitalized for COVID-19. In agreement with the aforementioned data, higher GDF-15 levels were associated with disease severity and mortality independently from traditional risk factors. Nonetheless, the present study first documented a trend toward a protective association between GDF-15 < 4.45 ng/mL and survival. Notably, the strength of association was descriptively more pronounced in patients with basal eGFR < 45 mL/min/1.72 m^2^. 

GDF-15 was herein inversely associated with renal function, being significantly higher in patients with eGFR < 45 mL/min/1.72 m^2^. The reasons for increased levels in the presence of reduced eGFR are debated. The low molecular weight hampers the plausibility of reduced clearance, suggesting increased renal synthesis and/or altered half-life as mechanisms responsible for its higher circulating levels in CKD [38]. Notably, present data suggests that the predictability of mortality risk in COVID-19 patients by GDF-15 could be stronger in the presence of eGFR < 45 mL/min/1.73 m^2^.

In the present study, GDF-15 emerged as the only biomarker independently associated with poor outcomes in non-dialysis renal patients affected by SARS-CoV-2 infection, out of the other 19 molecules responsive to inflammatory stimuli. Notably, the panel of cytokines, chemokines and uremic toxins that were investigated was built according to the literature review on the more promising biomarkers dysregulated in the course of COVID-19 and/or renal disease [52,53,54,55,56,57,58,59].

Present data needs to be taken cautiously due to several limitations: small sample size, the absence of pre-specified power calculation, the monocentric design and the advanced age of the enrolled population, which limits the generalizability of the results. Furthermore, a discrepancy between median values of eGFR in the whole cohort and the low prevalence of reported CKD make the baseline eGFR more susceptible to acute disease in addition to chronic renal damage. No data were available for discriminating contribution of renal, cardiac and pulmonary synthesis to GDF-15 circulating levels. The generalizability of the study deserves caution. The population enrolled had several differences compared with those usually reported in COVID-19 studies due to older age, lower BMI, absence of an invasive ventilation requirement and a low rate of steroid administration. Eventual, but not ascertained, limited life support might have influenced the value of prognostic markers in the present study. 

Taken together, these findings show that along with significant changes in inflammatory and cardiovascular biomarkers during SARS-CoV-2 infection, GDF-15 may represent a clinically useful risk stratification tool that provides important pathophysiological insights and prognostic information in CKD patients hospitalized with COVID-19. Specially designed studies are advocated to explore GDF-15 as the ideal candidate prognostic marker in the context of inflammatory diseases with pulmonary and cardio-renal involvement. 

## Figures and Tables

**Table 1:** (**A**) Patients characteristics. Demographics and comorbidities. (**B**) Patients’ characteristics. Clinical findings.

(A)				
Characteristic	All Patients	eGFR ≥ 45 mL/min	eGFR < 45 mL/min	*p*
	(*n* = 77)	(*n* = 44)	(*n* = 33)	
Sex. male	40 (52)	25 (57)	15 (46)	0.36
Age (years)	79 {70–86}	78 {69–86}	79 {73–86}	0.45
Ethnicity				
Caucasian	72 (94)	42 (96)	30 (91)	0.65
Middle east	1 (1)	0 (0)	1 (3)	0.43
Latin American	3 (4)	2 (5)	1 (3)	1.00
East Asian	1 (1)	0 (0)	1 (3)	0.43
BMI (Kg/m^2^)	25.9 ± 5.1	26.9 ± 5.3	23.9 ± 4.3	0.33
Medical history				
Hypertension	51 (66)	25 (57)	26 (79)	0.05
Cardio-vascular disease	38 (49)	20 (46)	18 (55)	0.49
Myocardial infarction	17 (22)	9 (21)	8 (24)	0.78
Heart failure	11 (14)	6 (14)	5 (15)	1.00
Arrythmias	15 (19)	9 (21)	6 (18)	1.00
Valvulopathies	4 (5)	3 (7)	1 (3)	0.63
Vascular disease	16 (21)	6 (14)	10 (30)	0.09
Cerebrovascular disease	8 (10)	7 (16)	1 (3)	0.13
Dementia	17 (22)	8 (18)	9 (27)	0.41
COPD	9 (12)	6 (14)	3 (9)	0.72
Asthma	1 (1)	1 (2)	0 (0)	1.00
Cancer	9 (12)	5 (11)	4 (12)	1.00
CKD	20 (26)	2 (5)	18 (55)	<0.001
Maintenance hemodialysis	4 (5)	0 (0)	4 (12)	0.03
Rheumatologic disease	2 (3)	1 (2)	1 (3)	1.00
Diabetes mellitus	25 (32)	10 (23)	15 (46)	0.05
Chronic liver disease	4 (5)	2 (5)	2 (6)	1.00
Age-adjusted CCI	3 {3–4}	3 {2–4}	3 {3–4}	0.58
**(B)**				
**Characteristic**	**All Patients**	**eGFR ≥ 45 mL/min**	**eGFR < 45 mL/min**	***p***
	**(*n* = 77)**	**(*n* = 44)**	**(*n* = 33)**	
Laboratory findings				
Hemoglobin (g/dL)	12.0 {11.2–13.3}	12.5 {11.4–13.8}	11.6 {10.7–12.6}	0.07
White blood cells (×10³/uL)	7.00 {5.42–9.71}	6.69 {4.93–9.03}	7.58 {6.01–10.22}	0.13
Neutrophils (×10³/uL)	4.93 {3.93–7.51}	4.67 {3.60–6.35}	5.87 {4.15–9.14}	0.05
Lymphocytes (×10³/uL)	1.04 {0.64–1.34}	1.05 {0.64–1.49}	1.01 {0.67–1.21}	0.61
N/L ratio	5.16 {3.12–9.81}	4.28 {2.89–8.20}	6.73 {3.77–11.47}	0.07
Platelets (×10³/uL)	204 {162–304}	222 {170–299}	192 {151–306}	0.52
C reactive protein (mg/L)	69 {27–99}	68 {27–102}	73 {27–98}	0.94
Procalcitonin (ng/mL)	0.13 {0.07–1.21}	0.11 {0.05–0.61}	0.21 {0.08–4.00}	0.315
Creatinine (mg/dL)	1.3 {1.0–2.2}	1.1 {0.7–1.2}	2.3 {1.6–3.8}	<0.001
Symptoms				
Fever	53 (69)	31 (71)	22 (67)	0.81
Anosmia/Disgeusia	3 (4)	2 (5)	1 (3)	1.00
Arthromyalgias	2 (3)	2 (5)	0 (0)	0.50
Cough	26 (34)	13 (30)	13 (39)	0.47
Dyspnoea	43 (56)	22 (50)	21 (64)	0.26
Abdominal pain	4 (5)	2 (5)	2 (6)	1.00
Nausea/vomiting	2 (3)	2 (5)	0 (0)	0.50
Diarrhea	3 (4)	2 (5)	1 (3)	1.00
Pneumonia on X-ray	67 (87)	38 (86)	29 (88)	1.00
P/F at admission	302 ± 99	297 ± 114	309 ± 77	0.96
SpO_2_ at admission	96 {91–97}	96 {90–97}	95 {93–97}	0.79
ARDS	36 (47)	19 (43)	17 (52)	0.50
Time (days)				
Symptoms ⟶ admission	4 {2–8}	4 {2–9}	5 {3–7}	0.88
Symptoms ⟶ Discharge/death	18 {11–35}	18 {11–29}	17 {11–52}	0.51
Admission ⟶ Discharge/death	12 {6–25}	10 {6–20}	16 {7–35}	0.07
Therapy				
Lopinavir/Ritonavir	10 (13)	6 (14)	4 (12)	1.00
Hydroxychloroquine	57 (74)	32 (73)	25 (76)	0.80
Remdesevir	1 (1)	1 (2)	0 (0)	1.00
Steroids	17 (22)	7 (16)	10 (30)	0.17
Heparin	58 (75)	33 (75)	25 (76)	1.00
Biological	10 (13)	4 (9)	6 (18)	0.31
Need for intensive care	2 (3)	1 (2)	1 (3)	1.00
Need for ventilation	42 (55)	25 (57)	17 (52)	0.65
Survivors	42 (55)	25 (57)	17 (52)	0.65

Abbreviations: BMI, body mass index; CCI, Charlson comorbidity index; CKD, chronic kidney disease; COPD, chronic obstructive pulmonary disease. Abbreviations: ARDS, acute respiratory distress syndrome; N/L ratio, neutrophil to lymphocyte ratio; P/F, ratio of arterial oxygen partial pressure to fractional inspired oxygen; SpO_2_, peripheral capillary oxygen saturation.

**Table 2:** Multivariate Cox regression model for survival in all patients, stratified by eGFR. Abbreviations: eGFR, estimated glomerular filtration rate; GDF-15, growth and differentiation factor 15; P/F, ratio of arterial oxygen partial pressure to fractional inspired oxygen.

Variable	All Patients		eGFR ≥ 45 mL/min		eGFR < 45 mL/min	
	HR (95% CI)	*p*	HR (95% CI)	*p*	HR (95% CI)	*p*
eGFR < 45 mL/min	0.58 (0.28–1.19)	0.14	-	-	-	-
Age ≥ 75 years	2.79 (1.19–6.59)	0.02	2.31 (0.75–7.09)	0.14	3.61 (0.95–13.79)	0.06
Fever	3.75 (1.38–10.17)	0.009	2.83 (0.63–12.74)	0.18	4.78 (1.17–19.56)	0.03
Dyspnea	1.78 (0.81–3.94)	0.15	2.22 (0.76–6.51)	0.15	1.21 (0.37–3.95)	0.75
P/F < 300	1.67 (0.82–3.41)	0.16	1.82 (0.65–5.09)	0.25	1.64 (0.56–4.77)	0.36
GDF-15. quartiles	2.28 (1.53–3.39)	<0.001	1.99 (1.17–3.39)	0.01	2.53 (1.34–4.79)	0.004

## Data Availability

Data are available on reasonable request.

## Supplementary Materials

The following supporting information can be downloaded at: https://www.mdpi.com/article/10.3390/biomedicines10123251/s1, Table S1: Association between all biomarkers and eGFR strata, mortality and need for ventilation; Table S2: Multivariate Cox regression model for survival predictability in all patients and in subgroups stratified according to eGFR, excluding outliers for GDF-15; Table S3: Multivariate Cox regression model for survival predictability in all patients and in subgroups stratified according to eGFR, excluding maintenance hemodialysis patients.
