# Supplementary material for: Growth Differentiation Factor 15 (GDF-15) Levels Associate with Lower Survival in Chronic Kidney Disease Patients with COVID-19
Source: Biomedicines. 2022 Dec 14;10(12):3251. doi: 10.3390/biomedicines10123251 (PMC9775159; doi:10.3390/biomedicines10123251)
Supplement: Supplementary file 1 [file biomedicines-10-03251-s001.zip › biomedicines-2081711-supplementary.pdf]

Supplementary Materials

Growth Differentiation Factor 15 (GDF-15) Levels for Predicting Mortality in Chronic Kidney Disease Patients with Covid-19

Andrea Galassi, Paola Ciceri, Valeria Bono, Lorenza Magagnoli, Matteo Sala, Luisa Artioli, Roberta Rovito, Mohamad Hadla, Vaibhav Yellenki, Antonella d'Arminio Monforte, Camilla Tincati, Mario Cozzolino and Giulia Marchetti

Table S1. Association between all biomarkers and eGFR strata, mortality and need for ventilation.

Molecules	eGFR	Mortality	Ventilation
**GDF-15**	*	****	*
**CD-25**	****	**	*
**RAGE**	*	****	***
**TNFα**	***	**	
**TNFR I**	****	***	
**TNFR II**	****	**	
**IL-7**	*	*	
**LIF**	***	*	
**FAS**	***		
**YKL-40**	**		
**IL-18**		**	***
**IL-6**		***	**
**PTX-3**		****	
**PDGF-AA**			
**IL-6R**			
**VEGF**			
**α1-AGP**			
**TRAIL**			
**Kynurenine**			
**Tryptophane**			

Caption: Cytokines levels (as continuous variable) were compared between eGFR strata, survived and deceased patients, and between patients who were and were not treated by non-invasive ventilation. Test for comparison was performed by Mann-Whitney U test (*p<0.05, **p<0.01, ***p<0.001, ****p<0.0001).

Legend:

α1-AGP: alpha 1-acid glycoprotein
eGFR: estimated glomerular filtration rate
GDF-15: growth and differentiation factor 15
IL-6: interleukine-6
IL-7: interleukin-7
IL-18: interleukin-18
IL-6R: interleukine-6 receptor
LIF: leukemia inhibitory factor
TNFα: tumor necrosis factor alpha
TNFR I: tumor necrosis factor receptor 1
TNFR II: tumor necrosis factor receptor 2
YKL-40PDGF-AA: platelet derived growth factor – AA
PTX-3: pentraxin-3 RAGE: receptor for advanced glycation end products
TRAIL: TNF-related apoptosis- inducing ligand kynurenine and tryptophane
VEGF: vascular endothelial growth factor

Table S2. Multivariate Cox regression model for survival predictability in all patients and in subgroups stratified according to eGFR, excluding outliers for GDF-15.

Outcome: mortality						
	All patients (no outliers)		eGFR ≥ 45 mL/min		eGFR < 45 mL/min	
Variable	HR (95% CI)	*p*	HR (95% CI)	*p*	HR (95% CI)	*p*
eGFR < 45 mL/min	0,66 (0,29-1,49)	0,3196	-	-	-	-
Age ≥ 75 y	2,96 (1,18-7,42)	0,0208	2,19 (0,70-6,83)	0,1769	6,02 (0,90-40,20)	0,0637
Fever	3,97 (1,36-11,62)	0,0117	2,69 (0,59-12,26)	0,2001	10,52 (1,29-85,44)	0,0277
Dyspnea	1,84 (0,79-4,27)	0,1549	2,08 (0,70-6,20)	0,1877	1,88 (0,36-9,97)	0,4575
P/F at admission < 300	1,54 (0,71-3,35)	0,2704	1,73 (0,61-4,94)	0,3027	1,39 (0,36-5,33)	0,6356
GDF-15, quartiles	2,25 (1,46-3,47)	0,0003	1,84 (1,07-3,17)	0,0281	3,68 (1,42-9,51)	0,0072

Caption:
eGFR: estimated glomerular filtration rate
GDF-15: Growth Differentiation Factor 15
P/F: PaO2/FiO2 ratio
Y: years

Table S3. Multivariate Cox regression model for survival predictability in all patients and in subgroups stratified according to eGFR, excluding maintenance hemodialysis patients.

Outcome: mortality						
	All patients (no HD)		eGFR ≥ 45 mL/min		eGFR < 45 mL/min	
Variable	HR (95% CI)	*p*	HR (95% CI)	*p*	HR (95% CI)	*p*
eGFR < 45 mL/min	0,65 (0,31-1,34)	0,2388	-	-	-	-
Age ≥ 75 y	2,31 (4,23-49,02)	0,0528	2,31 (0,75-7,09)	0,1447	1,80 (0,49-6,66)	0,3804
Fever	3,92 (1,42-10,80)	0,0083	2,83 (0,63-12,74)	0,1753	5,03 (1,14-22,27)	0,0334
Dyspnea	1,62 (0,73-3,58)	0,2328	2,22 (0,76-6,51)	0,1464	0,80 (0,24-2,67)	0,7174
P/F at admission < 300	1,70 (0,83-3,44)	0,1443	1,82 (0,65-5,09)	0,2537	1,47 (0,51-4,25)	0,4808
GDF-15, quartiles	2,28 (1,53-3,38)	4.49e-05	1,99 (1,17-3,39)	0,0114	2,87 (1,40-5,90)	0,0042

Caption:
eGFR: estimated glomerular filtration rate
GDF-15: Growth Differentiation Factor 15
P/F: PaO2/FiO2 ratio
Y: years
